# Open Lab: A web application for running and sharing online experiments

**DOI:** 10.3758/s13428-021-01776-2

**Published:** 2022-03-01

**Authors:** Yury Shevchenko

**Affiliations:** Research Methods, Assessment, and iScience, University of Konstanz, Universitätsstraße 10, D-78464 Konstanz, Germany

**Keywords:** Online data collection, Data management, lab.js

## Abstract

Online experiments have become a popular way of collecting data in the social and behavioral sciences. However, the high technical hurdles of setting up a server may prevent researchers from starting them. Also, proprietary software may restrict a researcher’s freedom to customize or share their study. Open Lab is a server-side application designed to host online surveys and experiments created using lab.js. Available online at https://open-lab.online, Open Lab offers a fast, secure, and transparent way to deploy studies; it handles uploading experiment scripts, customizing study design, managing the participant database, and working with the study results. Open Lab is integrated with the lab.js experiment builder (https://lab.js.org/), a browser-based program which enables the creation of new studies from scratch or the use of templates. This paper compares Open Lab with other study deployment services, discusses how Open Lab contributes to open science practices, and provides a step-by-step guide for researchers.

Social and behavioral scientists have collected data online since the earliest days of the internet (Gosling & Mason, 2015; Musch & Reips, 2000). As a place to implement traditional research methods, such as surveys, and explore novel internet-derived phenomena and methods, the internet itself has become a laboratory (Skitka & Sargis, 2006). The web browser has become a key technology—a way of delivering content and collecting responses in an experiment, although downloadable applications have also been used (e.g., Inquisit). Traditionally, web browsers were accessible via computer stations and laptops, but with the development of smartphones, mobile web browsers have also become research venues (with the difference being that creating and maintaining mobile applications is technically harder and often requires professional development). Building online experiments originally required enormous technical expertise, but it has become easier thanks to a variety of web-based resources specifically designed for cognitive psychologists (e.g., jsPsych, PsychoPy, lab.js). Nevertheless, study deployment by social scientists remains challenging since it requires yet another skillset and attention to data security. Moreover, because studies have often been specifically tailored to researchers’ study setups, they have been difficult to exchange.

Open Lab is a web application that makes hosting a study easy and provides a secure foundation for data collection. The application uses open-source code on GitHub and runs online at https://open-lab.online. Open Lab is directly integrated into lab.js, a web-based resource which enables the design and construction of an experiment in a web browser (Henninger et al., 2021). Indeed, Open Lab was designed to deploy lab.js experiments online in the most efficient way possible, with a focus on data security, high performance, and user experience. At the same time, Open Lab provides tools for collaboration between researchers, such as shared access to studies, the language localization of tasks, and the integration of data collection within the Open Science Framework (OSF).

Open Lab was launched in October 2018, and the first users were students testing the platform’s functionality. Within three years, the number of researchers using the platform increased, and more than 1000 studies have now been created^1^. During this time, we have improved the platform’s functionality, resolved errors, and refined the website’s visual design. We have also developed collaborative tools to support open science practices within the research community.

The present article outlines Open Lab’s features in comparison to other study deployment software. We discuss the open science tools built into Open Lab that can be used to improve the reproducibility of its studies. Finally, we provide a step-by-step user guide for researchers.

## Study deployment services

Once an experiment or a survey is created in the lab.js experiment builder at https://lab.js.org, a researcher faces the challenge of putting the study online and collecting data from participants. The lab.js builder supports various out-of-the-box ways—differing in ease of use and flexibility—for deploying studies from self-hosted servers to software. Previous research has reviewed a wide range of solutions for developing and hosting online studies, including lab.js (Sauter et al., 2020). Below, we focus on three main approaches for deploying a lab.js experiment: using a self-hosted server, integrating into proprietary software, and using open-source tools.

### Self-hosted server

Setting up a server from scratch is a relatively simple solution for experienced programmers or researchers who can easily get support from IT specialists. The server can be hosted on a university campus or rented from companies like Amazon Web Services, Heroku, Netlify, or DigitalOcean. The server’s configuration determines where and how the data will be stored, such as their location, encryption, and backup schedule.

Regarding the server programming language, the lab.js builder supports integration with a PHP server, which remains a popular server-side web language^2^. Another way to host one’s own server is with Netlify’s service, which can connect to a Git repository (e.g., GitHub, GitLab) and offers a free basic plan for personal projects.

Setting up a dedicated server enables full control over a project and maximum freedom, but it requires expertise and regular maintenance. In most cases, there is no graphical user interface, so researchers must work with the command line. Moreover, the data should be managed, organized, and stored so as to ensure protection against security breaches. Thus, a self-hosted server might be burdensome for a researcher who prefers to focus on the more significant research matters of study design and data analysis.

### Integration into proprietary software

A lab.js study can be integrated with proprietary software such as Qualtrics or SoSci Survey. Although many of these services have been designed for surveys, they may contain JavaScript code. The advantages of proprietary software are user technical support and ready-made solutions that simplify the workflow. Research groups that have already used proprietary software will find adding a lab.js experiment to their existing studies easier.

In contrast, the price of a proprietary service may be too high for an independent researcher. For example, Qualtrics’ annual plan starts at USD 1500 per year, according to online reviews^3^. Moreover, since these commercial tools have mainly been built for questionnaire research, they often require a great amount of customization to get a lab.js experiment running. In Qualtrics, researchers still need to host a lab.js study externally and use an *iframe* to embed it in the Qualtrics survey.

A lab.js experiment can also be integrated into the Pavlovia data collection platform. Pavlovia supports studies built using PsychoPy, jsPsych, and lab.js, and it uses the Git version control system to manage projects. As described on Pavlovia’s website^4^, although there is no charge for running a pilot test, conducting a full study requires the purchase of participant credits (GBP 0.20 per participant) or an institutional license (GBP 1500 per year, with no participant limit).

### Open-source tools

Regarding the amount of effort and resources required, open-source software occupies the middle ground between a self-hosted server and proprietary software. Open-source tools can simplify a researcher’s workflow, but they still require them to come to grips with technical details and terminology in order to configure those tools. Researchers often develop open-source software with a very concrete intention, limiting it to a very specific purpose. With this in mind, Open Lab has been developed for integration with lab.js, and it does not support experiments created in other software, such as jsPsych or PsychoPy^5^.

As an alternative to Open Lab, researchers have the options of using JATOS or the Experiment Factory to deploy a lab.js experiment. *JATOS*, or “Just Another Tool for Online Studies,” is a JAVA application that can be installed on a web server to host online studies (Lange, Kühn, & Filevich, 2015). JATOS is intended to work across platforms and support any experiment scripts written in JavaScript. Once installed on a server, JATOS provides a graphical user interface for participant management and data collection^6^. The Experiment Factory uses *docker* containers to encapsulate all the dependencies and static files required for an experiment (Sochat, 2018). The container can be integrated with the local file system to save collected data or connected to a database such as SQLite, MySQL, or PostgreSQL^7^. Both JATOS and the Experiment Factory can be a preferred solution for researchers with JAVA or *docker* skills and offer more flexibility in conducting any JavaScript-based experiments.

Open Lab is open-source software. The code is available on GitHub^8^. Research institutes that require data storage on their local servers can install and use the Open Lab application at their locations. Yet Open Lab is also available as a ready-to-use web platform at https://open-lab.online. The platform has a free plan that includes the ability to conduct a study involving up to 300 participants. That limit can even be exceeded if the data are stored in the OSF, with which Open Lab provides seamless integration. Thus, Open Lab can support most research projects by providing a fast start for online data collection while removing technical obstacles along the way.

For larger research projects and laboratories, the platform offers a paid subscription with a maximum cost of EUR 20 per month (about USD 25). This subscription model ensures the platform’s sustainability and enables its developers to cover the costs of further technological development, web hosting, and regular server backups^9^.

## Open science practices

What defines open data or content is that it “can be freely used, modified, and shared by anyone for any purpose”^10^. Open *science* allows people to collaborate and contribute in ways that “enable reuse, redistribution and reproduction of the research and its underlying data and methods”^11^. “Open science” is not only the concept of knowledge accessibility or collaborative research, but also a supporting technological infrastructure (Fecher & Friesike, 2014).

Open Lab fosters open science practices by being a way to archive, share, and customize experiment scripts on the same platform. These features facilitate collaboration among researchers and improve the reproducibility of research. In general, reproducibility can be represented at different levels—for example, computational reproducibility (calculating the same results from the same data), direct experimental reproducibility (obtaining the same results from a standardized experiment), or indirect experimental reproducibility (obtaining the same results from different experiments) (Leonelli, 2018). Although there has been much discussion about computational reproducibility and the ability to create and curate durable databases, experimental reproducibility remains problematic as replications generally provide weaker evidence for the original results (Aarts et al., 2015).

One reason for the difficulties with replication is that researchers sometimes use different software for the same experimental paradigms. For example, there may be a dozen versions of the Stroop Task programmed in different software or many variations of this task in one type of software. Different software can introduce slight variations in experimental design that are hard to control for without knowing the specifics of each software. Heterogeneous data collection methods themselves might not be so problematic, as they allow indirect replication, i.e., obtaining the same results from a variation in an experimental paradigm. However, the problem of heterogeneity is exacerbated by the lack of code transparency when data collection methods are reported. Converting a natural language description of an experiment, which almost inevitably contains some ambiguity, into computer code may lead to discrepancies (Ince, Hatton, & Graham-Cumming, 2012). This becomes clear when researchers try to replicate published studies: journal articles or supplementary materials rarely provide the level of precision necessary to reproduce an experiment without the need to contact the authors to obtain missing details or clarify uncertainties.

Although computational reproducibility has progressed with the support and promotion of open-access data, the same effort should now be made to provide open access to data collection methods. For example, study protocols and open-source experiment code can be linked to publications. Protocols can be informal (e.g., screenshots, video recordings) or formal, following standards such as DRESS (Documenting Research in the Empirical Social Sciences), a protocol developed by the TIER project^12^. Providing open access to experiment scripts seems more straightforward in online research than in laboratory research, since the experiment’s code is already shared on the internet during data collection. For example, the Experiment Factory aims to utilize reproducible containers that capture all experiment dependencies and run them at any time in the future (Sochat, 2018).

lab.js addresses code transparency and reproducibility problems by using scripts that are saved as JSON files and can be inspected and run again at any time. Each lab.js script uploaded to the Open Lab platform is called a “task” and represents an experiment or a survey. Open Lab can combine several experiments or surveys into one study.

Open Lab provides tools for the collaborative sharing of studies and differentiates between a study’s accessibility to participants (whether the study is in development or ready for data collection) and its accessibility to researchers (whether the study is private, shared with a group of researchers, or public). Table 1 describes the differences between different study statuses.

**Table 1:** Study statuses on Open Lab

	Private	Shared	Public
Inactive study	Study in development	Study in collaborative development	Archiving, sharing, and meta-analysis
Active study	Data collection	Collaborative data collection	Collaboration between research groups

*Private* studies are not displayed to website visitors. Data collection is still possible, however, if participants are invited to do so using a special study link. Thus, Open Lab imposes no obligation to disclose a study during its development phase or while researchers wish to keep their experiment’s code private.

*Shared* studies make their tasks, task parameters, participants, and results accessible to collaborators and enable collaborative study development or data collection. A study’s author has more rights than invited collaborators, such as the right to edit or delete the study; invited members cannot access these options. However, collaborators have access to all other functions: opening a study to the public or to invited participants only, selecting tasks, customizing parameters, sending invitations, and downloading results. Within the study project, collaborators can review and archive the raw data as soon as a participant has completed a task (see, for example, “born-open data,” Rouder, 2016). Sharing access to studies fosters collaboration between researchers and enables, for example, working on the task in different languages.

**Fig. 1:**
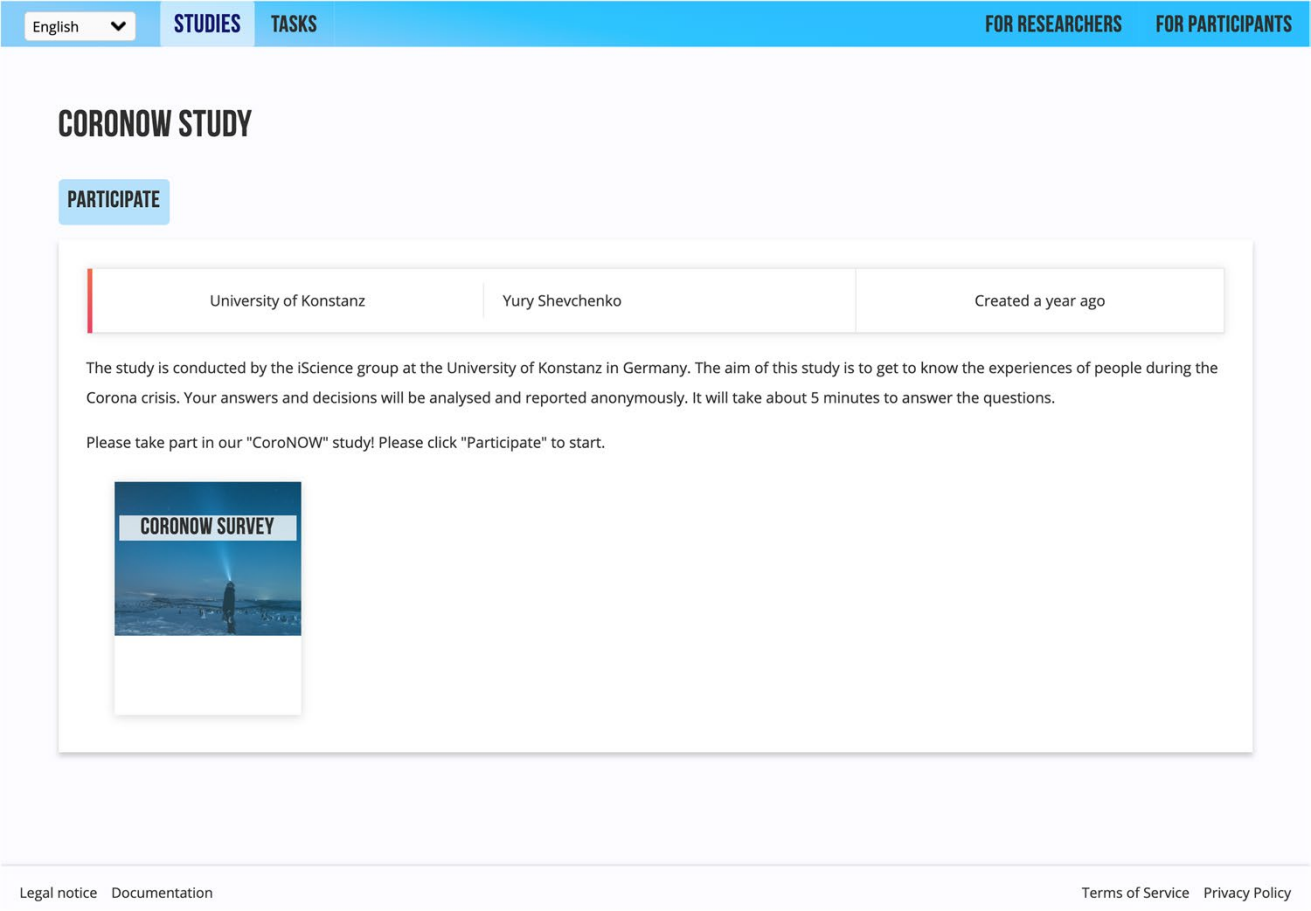
Screenshot of an Open Lab study home page

*Public* studies are visible to all the website’s visitors in its library of public studies. These published studies, or parts of studies, should help to give researchers an identity and enhance their reputations, which is another requirement for open science infrastructure (Leible, Schlager, Schubotz, & Gipp, 2019). Such systems link researchers to their contributions, and they receive credit for the efforts they have made and the value they have created. A public study format can be used to collect data, while the study is still active, or as an archive for completed studies, which are available for further inspection and meta-analysis, once studies have become inactive. A public study has a dedicated web page with a unique URL address (see Fig. 1). When the study is active, the link for participants is displayed on the page. Once the study has been archived, the link is removed from the page, but the study description and experiment scripts remain publicly available. For data security reasons, it is impossible to participate in another study using a researcher account on Open Lab. This is done to prevent any malicious scripts that may have been embedded in a lab.js script from accessing the researcher’s data. To inspect a study’s code or run the study itself, a researcher can open it in the lab.js builder.

Open Lab also provides sharing functionality for individual tasks. Like studies, tasks can be private, shared, or public. *Public* tasks can be shared in two ways: as a reference and as a full copy. Referencing is done via the Open Lab interface by adding a public task to the study. If the author later updates the task, it will also be updated in all the studies using that task. Furthermore, any task can be customized with study-specific parameters. Sharing the task by reference is useful if several research groups are working on a project in which the same version of the task is tested in different laboratories, such as the Many Labs project (Aarts et al., 2015). On the other hand, a full copy of the task (available via the lab.js builder) allows a researcher to add an independent copy of the task to a different project. Therefore, the ability to create an independent copy of the task can be used for task adaptation and more extensive customizations, which can be done by editing the code in the lab.js experiment builder.

## Step-by-step guide for researchers

The subsections below explain how Open Lab works from the perspective of researchers and participants. First, we describe the steps required to upload a task from the lab.js builder, we address the process of creating and managing the study, and we explain how to work with collected data. We also describe how to use the website from the participant’s point of view. Open Lab’s technical specifications are given at the end.

### Researcher workflow

#### Uploading a task

**Fig. 2:**
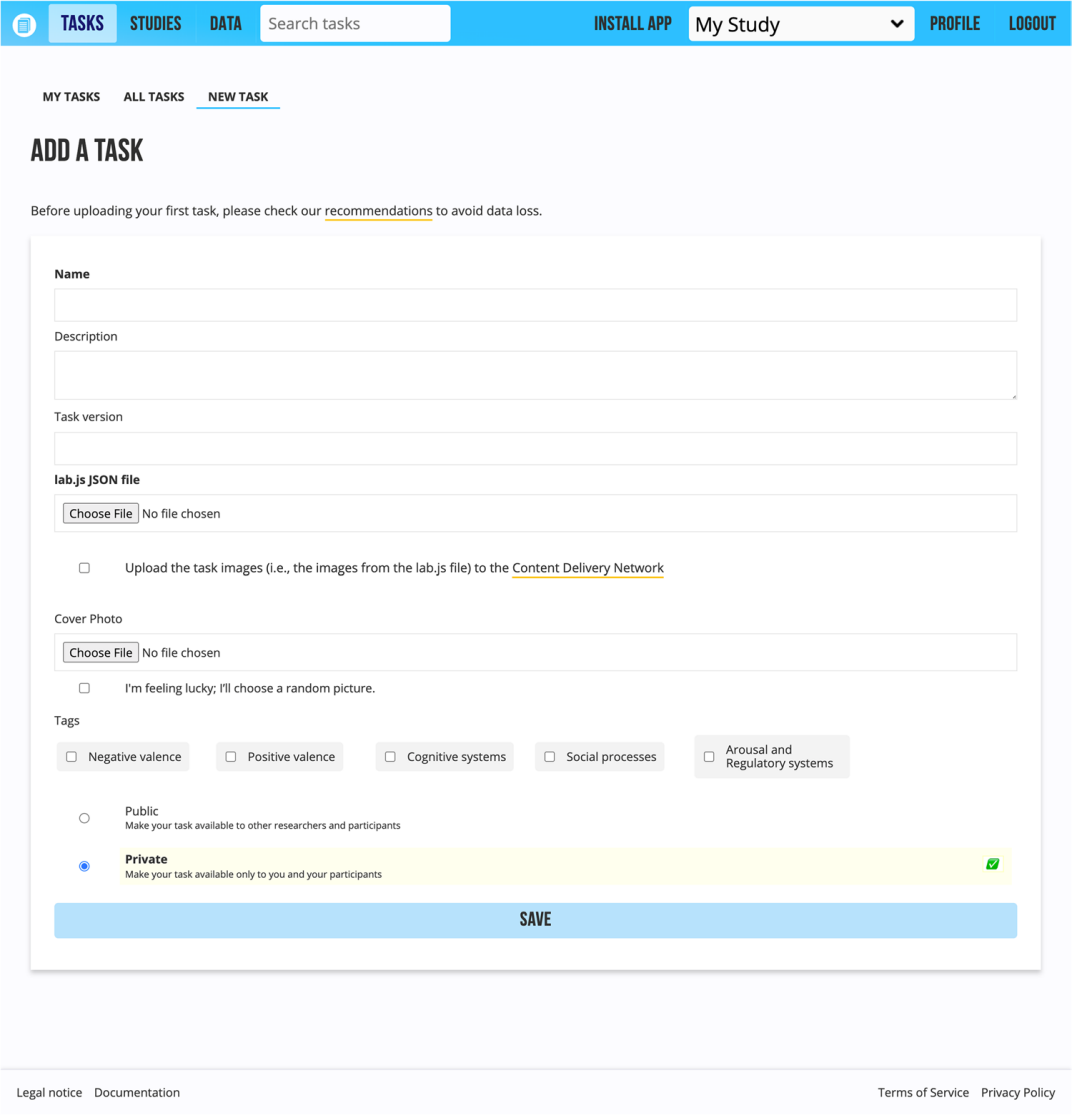
The New Task Upload Form

There are two ways to upload a lab.js script to Open Lab. First, researchers can deploy the task directly from the lab.js builder by selecting “Upload to Open Lab” in the export menu. Second, they can upload the task as a JSON file from the “New task” page (under the “Tasks” tab). In both cases, the form for adding a new task contains task information such as a name, description, task version, cover image, tags, and privacy settings (see Fig. 2). The new task can be private (available only to researchers and invited participants) or public (available to all website visitors).

Open Lab provides a quick path between task development in lab.js and deployment in Open Lab. A task in the lab.js builder can be exported to Open Lab with a few clicks. Similarly, any task in Open Lab can be opened with one click in the lab.js builder.

#### Setting up a study

Open Lab allows tasks and studies to be separated so that a single study can contain many tasks and a task can be used in many studies. A researcher can create a new study from the “Studies” page by giving it a name and a description. The study can be shared with colleagues by providing their email addresses. If required, a confirmation code can be displayed at the end of the study (which participants can enter as proof they have completed the study on their recruitment website, such as Amazon Mechanical Turk) or participants can be redirected to an external web page. Once a study has been created, tasks can be added to it via the “Select tasks” page. The “Invitations” page contains web links for the participants: Open Lab supports different authentication strategies, such as a participant code, email, or an external social network account^13^. The study can be tested via the “Try demo” page. Researchers’ results are kept separate from participants’ results and are available on the “Demo results” page.

Open Lab also supports between-subject study designs via a randomization of both tasks and parameter values. Those features are explained in more detail in the website documentation. A researcher can customize task parameters without needing to edit the original lab.js experiment script in the builder. To enable this, a sequence component has to be included in the lab.js builder as a parent for all the experiment components. Parameters that are shared across the experiment, such as the number of trials or the stimulus presentation time, must be defined for this parent component. Once the task has been uploaded, these parameters are available for editing via the Open Lab interface.

#### Collecting data

Participants’ data can be accessed from the “Data” page. Data can be exported as CSV or Excel files and filtered by studies, tests, or participants. They can also be downloaded together or as separate completed tasks. For data safety, Open Lab transfers the data to the server incrementally during the task (“incremental data”) and sends the complete dataset once again at the end of the task (“full data”). Saving incremental data prevents data loss if a participant interrupts a task midway through it, and saving full data prevents data loss due to unstable internet connections during the task. Researchers can either download all data (both incremental and full) or only the full data from completed tasks. It is also possible to download participants’ metadata only (e.g., their device type, browser version, screen width)^14^.

An Open Lab study can be linked to a new or existing project on OSF. Once this is done, as soon as a participant completes a task on Open Lab, all the data will automatically be sent to and saved in the OSF project.

### Participant workflow

Open Lab provides a variety of authentication strategies for different categories of participants. For one-time users, a unique, random, ad hoc participation code is created. Alternatively, users can enter a participation code themselves or use their email or social network account to sign up. Users’ credentials, such as their social network ID, are only used for authentication purposes and are not visible to researchers.

User authentication can leverage several benefits: participants can take part in many studies and have access to their results; furthermore, researchers can interact with participants by giving them feedback, conducting debriefings, inviting them to other studies, or running multiple experiments separated in time.

After authentication, the participant sees a task flow displayed on a dashboard showing which tasks have been accomplished and which task comes next. Users cannot participate in the same task twice if they have already completed it (in cases where the researcher activates this feature). Once all the tasks in a particular study have been finished, users see a confirmation code that both they and researchers can use to verify their participation. Participants can take part in different studies by switching between them on a page with the list of active studies. This list is a good place for researchers to promote new studies.

In accordance with the General Data Protection Regulation (GDPR) data protection guidelines, participants have the right to know what data pertaining to them have been collected during an experiment and are available to researchers. Open Lab also gives participants the opportunity to request that their data be deleted. In such case, the researcher will see that the participant’s data have been marked with a special flag. It is then the researcher’s responsibility to ensure that the data are deleted.

If data collection happens in a laboratory, Open Lab can be installed as a web application on a desktop computer (the “Install app” link is displayed in the top navigation bar). Progressive web application technology makes the application accessible via an icon and removes browser features from its interface, such as the “Back” button.

### Technical specifications

In technical terms, Open Lab is a server-side Node.js application written in JavaScript. It can be installed on a server (e.g., Apache) via *node package manager* (npm), which manages all the dependencies listed in a *package.json* file. A process management tool (e.g., PM2) is recommended for running the application on the server. The application’s server logic is implemented through the Express.js framework. All data are stored in MongoDB databases, which can be installed on the same or a different server. All the details and installation instructions for setting up a custom server can be found on the GitHub project page. The project running service at https://open-lab.online is accessible via modern browsers such as Mozilla Firefox, Chrome, Safari, and Opera.

The data in Open Lab are not stored in a file system but rather in a separate MongoDB database that runs on the server (or it can be set up on a different server if required). The server is isolated by a firewall, and the database is configured to communicate only with the web application on the server. Tasks and results are stored in different database repositories so that a task’s public exposure is not associated with the results. Only researchers with shared access to a study can view its results.

Open Lab supports both continuous data transfer and transfer of the complete data at the end of a task. Since the experiment script runs on the client side, data transfer should be consistent throughout the experiment but should not interfere with the running task. Therefore, the latest data are sent to the Open Lab server if no events occur within 2.5 seconds. This method saves incremental data even if a participant drops out before the end of a task. A researcher can use these data to analyze whether a dropout was associated with an experimental condition, thus excluding a potential threat to the task’s validity (Arechar, Gächter, & Molleman, 2018). Complete data are also transferred to the server at the end of the task.

## Conclusion

Open Lab makes the task of deploying an online study an easy one for researchers. Open-source code allows the code to be used or contributed to for its development. Any researcher who needs to use Open Lab can use the service running at https://open-lab.online, the site that hosts online experiments designed in the lab.js experiment builder. Open Lab promotes the open science practices of sharing methods and raw data between researchers and inspires collaboration between different laboratories.

## Footnotes

1 As of December 2021, 1150 studies with at least 10 participants have been conducted on the Open Lab platform, and the total number of participants was about 90,000.

2 The latest lab.js documentation for PHP and other deployment strategies is available at https://labjs.readthedocs.io/en/latest/

3 Qualtrics’ pricing is not publicly available on its website https://www.qualtrics.com/, so the price was estimated from online reviews, e.g., https://uk.pcmag.com/cloud-services/118019/qualtrics or https://www.quora.com/What-is-Qualtrics-cost

4 https://pavlovia.org/docs/credit-license/overview

5 Integration of Open Lab with other JavaScript-based experimentation tools is feasible and may be implemented in the future.

6 JATOS documentation can be found at https://www.jatos.org/. JATOS also offers a free server MindProbe for hosting online experiments at https://mindprobe.eu/.

7 User and developer guides with example of experiments can be found at https://expfactory.github.io/

8 https://github.com/Yury-Shevchenko/openlab

9 The Open Lab platform is legally represented by Open Lab Online UG (haftungsbeschränkt), a company registered in Germany. All data are stored on servers in Germany according to the EU’s General Data Protection Regulation (GDPR). The privacy policy can be found at https://open-lab.online/docs/policy, terms of service can be found at https://open-lab.online/docs/terms, and our legal notice can be found at https://open-lab.online/docs/legalnotice.

10 https://opendefinition.org/

11 https://www.fosteropenscience.eu/foster-taxonomy/open-science-definition

12 https://www.projecttier.org/tier-protocol/dress-protocol/

13 Open Lab studies can be integrated with other data collection software (e.g., Qualtrics, SurveyMonkey, formr.org, etc.) by using redirects with query strings. Documentation is available at https://open-lab.online/docs/project#integration

14 Documentation about collecting participants’ metadata is available at https://open-lab.online/docs/data#metadata.

## Declarations

### Code availability

The Open Lab application has an open-source code on GitHub (https://github.com/Yury-Shevchenko/openlab) and is available online at https://open-lab.online.
